# A Randomised Controlled Trial of Efficacy of Cognitive Rehabilitation in Multiple Sclerosis: A Cognitive, Behavioural, and MRI Study

**DOI:** 10.1155/2016/4292585

**Published:** 2016-12-27

**Authors:** J. Campbell, D. Langdon, M. Cercignani, W. Rashid

**Affiliations:** ^1^Clinical Imaging Sciences Centre, Brighton and Sussex Medical School, Falmer, UK; ^2^Department of Psychology, Royal Holloway, University of London, London, UK; ^3^Department of Neurology, Brighton and Sussex University Hospitals NHS Trust, Brighton, UK

## Abstract

*Aim.* To explore the efficacy of home-based, computerised, cognitive rehabilitation in patients with multiple sclerosis using neuropsychological assessment and advanced structural and functional magnetic resonance imaging (fMRI).* Methods.* 38 patients with MS and cognitive impairment on the Brief International Cognitive Assessment for MS (BICAMS) were enrolled. Patients were randomised to undergo 45 minutes of computerised cognitive rehabilitation using RehaCom software (*n* = 19) three times weekly for six weeks or to a control condition (*n* = 19). Neuropsychological and MRI data were obtained at baseline (time 1), following the 6-week intervention (time 2), and after a further twelve weeks (time 3). Cortical activations were explored using fMRI and microstructural changes were explored using quantitative magnetisation transfer (QMT) imaging.* Results.* The treatment group showed a greater improvement in SDMT gain scores between baseline and time 2 compared to the control group (*p* = 0.005). The treatment group exhibited increased activation in the bilateral prefrontal cortex and right temporoparietal regions relative to control group at time 3 (*p* < 0.05_FWE  corrected_). No significant changes were observed on QMT.* Conclusion.* This study supports the hypothesis that home-based, computerised, cognitive rehabilitation may be effective in improving cognitive performance in patients with MS. Clinical trials registration is ISRCTN54901925.

## 1. Introduction

Cognitive impairment is present in 40–65% of individuals with MS [1]. Studies have shown cognitive deficits (in particular deficits in information processing speed, concentration, and working memory) to be present in the early stages of MS [2–4]. Cognitive impairment has a negative impact on quality of life (QOL) independent of physical symptoms [5, 6]. 

There exists mounting evidence for neuroplasticity as a mechanism to compensate for accumulating pathology in MS and some tentative evidence that cognitive rehabilitation may be effective in preserving or improving cognitive function in patients with MS [7–9]. Computer-assisted cognitive rehabilitation has the potential to provide a structured and standardised approach to rehabilitation. RehaCom is one particular type of software designed and utilised for treatment of cognitive impairment in a number of disease states such as stroke, brain injury, and psychiatric disorders [10, 11]. It has been used in a growing number of trials of cognitive rehabilitation in MS as a more standardised intervention [8, 9, 12, 13]. The difficulty level of the computerised tasks adapts to an individual's performance, only increasing in difficulty in response to improving performance.

Few studies have examined the structural basis of cognitive rehabilitation and longitudinal studies are relatively lacking [8, 14]. Animal data suggest that myelination is, at least in part, regulated by neuronal activity [15]. It is therefore conceivable that techniques, such as magnetisation transfer (MT), which is sensitive to myelin content, might be sensitive to structural plasticity in MS [16].

In this study we combined neuropsychological assessment, functional MRI (fMRI), and quantitative magnetisation transfer (QMT) imaging to explore whether home-based, computerised cognitive rehabilitation is an effective means of promoting cognitive rehabilitation and whether the structural basis for rehabilitation can be better defined [17]. The primary outcome of the study was measured as any improvement in cognition after the training, while the secondary outcomes included changes in fMRI, fatigue, and quality of life assessments.

## 2. Subjects and Method

*Participants*. Thirty-eight patients with objective evidence of cognitive impairment were invited to participate in this study between February 2014 and February 2015. All participants signed informed written consent before undergoing testing. Inclusion criteria were as follows: (a) age between 18 and 65, (b) clinically definite MS, according to the McDonald criteria [18], (c) Expanded Disability Status Scale (EDSS) ≤ 6.5, and (d) cognitive impairment defined as scores below the 5th percentile for normative data adjusted for age, sex, and years of formal education [19] on one or more of the BICAMS tests.

Patients were excluded if they had a history of significant psychiatric disorders, alcohol or substance abuse, visual acuity less than 6/18 corrected, oscillopsia, or diplopia that would interfere with testing. Patients were also excluded if they had a MS relapse, received corticosteroids, or changes made to psychoactive medications within the previous month.

The study was approved by the Northern Ireland Research Ethics Committee.

*Study Design*. An open-design, randomised, controlled trial was conducted. Neuropsychological and MRI data were obtained at baseline (time 1), immediately following a 6-week intervention period (time 2) and after an additional 12-week follow-up period (time 3), during which no additional intervention was administered (supplementary Figure 1 in Supplementary Material available online at http://dx.doi.org/10.1155/2016/4292585).

It was not possible for the cognitive assessments to be completed by a blinded assessor. The MRI analysis was conducted by a researcher blind to the patients' group allocation.

*Cognitive and Behavioural Assessments*. At entry all participants underwent a detailed clinical neurological assessment including EDSS conducted by an experienced neurologist. Patients were screened for cognitive impairment using the Brief International Cognitive Assessment for MS (BICAMS). BICAMS is a brief (15-minute) screening tool to identify cognitive impairment in patients with MS and comprises the first five learning trials of the California Verbal Learning Test II (CVLT-II), the first three recall trials of Brief Visuospatial Memory Test Revised (BVMT-R), and the Symbol Digits Modalities Test (SDMT) [20].

The BICAMS assessment was conducted by a neurology clinical fellow with almost ten years of clinical experience (J.C). The assessing neurologist was trained in BICAMS assessing methods by an experienced neuropsychologist (D.L).

At baseline participants also completed a number of behavioural and QOL assessments including EuroQOL five-dimension questionnaire (EQ-5D), a generic health-related quality of life scale [21], Functional Assessment of MS (FAMS) (a MS specific quality of life scale) [22], Patient Activation Measure (PAM-13) (a 13-item generic scale for chronic illness management), a measure of patient “empowerment” in MS [23], Unidimensional Self-Efficacy scale for MS (USE-MS) [24], the Hospital Anxiety and Depression Scale (HADS) [25], Multiple Sclerosis Neuropsychological Questionnaire (MSNQ) self-report (a patient self-reported measure of cognitive function) [26], and the Fatigue Severity Scale [27].

At each subsequent time point participants underwent repeat cognitive assessment using BICAMS (same test forms) as well as repeat behavioural and QOL assessments.

*Randomisation*. Following baseline MRI, patients were randomised to either the treatment or control groups. Randomisation was performed using a random number generator and allocations were placed inside sealed folders. Folders were opened following baseline MRI.

*Intervention*. The treatment group underwent six weeks of home-based, computer-assisted cognitive rehabilitation using RehaCom software (https://www.fixxl.co.uk/). This consisted of 45-minute sessions, three times weekly. The control group were asked to watch a series of natural history DVDs of corresponding duration and frequency to the rehabilitation sessions performed by the treatment group for six weeks. The need to evaluate MRI parameters in studies with active control conditions has been highlighted [14].

Treatment sessions consisted of training in three specific modules involving working memory, visuospatial memory, and divided attention. In all tasks the level of difficulty is tailored to the individuals performance and increases automatically but only in line with satisfactory progress. Real-time data pertaining to performance, progress, and compliance is transmitted to the investigator over the Internet during the intervention period.

*“Divided Attention” Module*. In the divided attention task the patient is asked to drive a simulated car using keyboard inputs. Multiple distractions must be navigated and the speed and direction of the vehicle altered according to road conditions. As the complexity of the task increases, more distractors are introduced with increased multitasking skills required.

*“Working Memory” Module*. The working memory task consists of remembering a series of playing cards presented briefly on screen. The participant is then asked to select which cards were presented from a longer series of options including distractor cards. As the complexity of the task increases, participants are asked to remember only cards of a particular value or suit and the number of items to remember increases. Higher levels involve having to remember the cards in reverse order.

*“Topological Memory” Module*. Visuospatial memory is a similar task involving various objects presented briefly on screen with the patient asked to remember the object as well as its position in the sequence. As the complexity of the task increases, the number of items on screen increases and more abstract shapes are introduced.

*MRI Imaging Protocol*. The following sequences were acquired in an order designed to minimise the potential for fatigue on the fMRI task: (1) dual-echo turbo spin-echo for lesion identification; (2) high-resolution T1-weighted magnetisation-prepared rapid-acquisition gradient echo (MPRAGE); (3) functional MRI with echo-planar imaging (EPI) acquired during a *n*-visually presented back task; (4) quantitative magnetisation transfer (QMT) with balanced steady-state free precession (bSSFP) [28].

T2 lesion volume was measured at baseline for each participant using the software package JIM (Version 3.0, Xinapse Systems Ltd., Northamptonshire, UK, http://www.xinapse.com/).

*N-Back Task*. A visual *n*-back test was presented during functional imaging acquisition. This was adapted from Sweet et al. [29] and involved three conditions: 0-back, 1-back, and 2-back tasks. The 0-back condition was designed to act as the baseline condition and would provide the baseline activation for comparison in fMRI analysis. The 1-back and 2-back conditions provided increasing working memory demands.

The *n*-back task did not constitute part of the cognitive rehabilitation. All participants were allowed to briefly practice the *n*-back task under supervision for five minutes prior to the MRI scan to ensure comprehension of the task and allow familiarity with it.

The visual *n*-back task was presented using Cogent V and MATLAB 2013a. Stimuli were projected onto a mirrored screen inside the MRI scanner 45 cm from a participant's nose. An MRI compatible button box was placed in the participant's right hand.

White letters were projected onto a black background in bold size 200 Arial font. This involved of a series of pseudo-randomised consonants in both upper and lower cases. The stimulus duration was 1000 ms with a between stimulus interval of 2000 ms. Instructions were presented for 3000 ms before each new *n*-back task.

fMRI data were acquired during three 9-minute runs. 0-back, 1-back, and 2-back tasks were presented in a randomised manner resulting in six blocks per nine-minute run. Each block consisted of 126 stimuli, one-third of which were targets. Twice as many 0-back tasks were presented as 1-back or 2-back. There was a rest period of 90 seconds between blocks.

*Statistical Analysis*. The primary outcome was cognitive performance as measured by improvement in SDMT, BVMT, and CVLT between groups compared to baseline. Secondary outcomes were QOL, fMRI, and QMT measures as detailed below.

*(i) Behavioural Data*. Descriptive statistics for normally distributed continuous variables are expressed as mean and standard deviation. Skewed continuous variables were summarised using median and interquartile range (IQR). Categorical variables are summarised by frequencies and percentages.

Normality of continuous variables was assessed using the Kolmogorov-Smirnov test.

Baseline cognitive and behavioural measures were compared between the treatment and control groups. Categorical variables were compared by the Pearson *χ* ^2^ test. The means of continuous variables were compared using the independent samples *t*-test or the Mann–Whitney *U* test for skewed data.

All tests were two-tailed; *p*-values less than 0.05 were considered significant.

Outcomes were compared between the two groups using independent samples *t*-test to compare gain scores for cognitive data between groups. To compare differences between groups for other behavioural and QOL data, a 2 × 3 repeat measures analysis of variance (ANOVA) was used with “time” as the within-subject factor and “treatment” as the between factor (active rehabilitation versus control).

Analyses were performed using SPSS version 21 (Armonk, NY: IBM Corp).

*(ii) Functional MRI Analysis*. fMRI data were analysed using SPM8 (Wellcome Department of Cognitive Neurology, UCL, London, http://www.fil.ion.ucl.ac.uk/spm/).

For each time series, the first five EPIs were discarded to ensure steady-state magnetisation. Individual EPIs were then realigned to the first remaining image of the series by rigid-body transformation to correct for involuntary head movements during acquisition before normalisation into a standard anatomical space (Montreal Neurological Institute [MNI]) using linear and nonlinear transformations. Finally, images were smoothed with an 8 mm^3^ full-width-at-half-maximum (FWHM) 3D Gaussian kernel.

*First-Level Analysis*. For each participant, the difference in blood oxygen level-dependent (BOLD) response between the 0-back, 1-back, and 2-back conditions was estimated at every voxel across the whole brain using the general linear model (GLM). This produced a series of contrasts representing mean activation during each *n*-back condition minus the 0-back condition.

*Second-Level Analysis*. Each contrast obtained at the first-level was entered into a second-level GLM to generate summary statistical parametric maps (SPMs). For between-group analysis of difference between the time points, we used a 3 × 2 ANOVA flexible factorial design with group (between-subject) and time (within-subject) as separate factors to examine the main effects on group (treatment versus control), time and the interaction between them to evaluate areas of relative change in activity after cognitive training versus control.

The threshold for significance was set at alpha of 0.05 corrected for multiple comparisons (family-wise error (FWE) corrected). Results are reported at cluster level throughout. Within each region of statistical significance, the location of local maxima of signal intensity increase is expressed as *x*, *y*, and *z* coordinates in MNI space.

*(iii) Quantitative MT Analysis*. The MT data were analysed using SPM8. MT and T1 mapping data from all three sessions were first realigned to subject specific MPRAGE structural images using the SPM8 rigid-body registration function. The MPRAGE were then segmented into white matter, grey matter, and CSF to yield a parenchymal mask.

A T1 map was calculated for all datasets by fitting the theoretical spoiled gradient echo as a function of the flip angle to the signal measured by the 3D FLASH sequences [30]. MT parameters were obtained by performing a voxelwise nonlinear least squares fitting (Levenberg-Marquardt) to a binary spin bath model for bSSFP.

The statistical analysis was performed voxelwise in SPM8 on the resulting warped and smoothed MT maps. The same GLMs used for the second-level fMRI analysis and described in the previous section were used for estimating the main effects of time and group and the interaction between these two factors.

## 3. Results

*Baseline Characteristics*. 38 patients were included in the study. The majority of the participants were female (71.1%). At entry 27 patients (70.3%) had RRMS, and 11 patients (29.7%) had SPMS. Patients were aged between 32 and 62 (mean 47.37, SD 8.23). The duration of MS from diagnosis to enrolment ranged from 12 months to 40 years (mean 11.61 years, SD ± 8.2 yrs). Median EDSS was 5.0 (3.5–6.0). 20 patients (52.6%) were on disease modifying therapy at enrolment (natalizumab *n* = 6, beta-interferon *n* = 7, fingolimod *n* = 6, and teriflunomide *n* = 1).

After randomisation to either computer-assisted cognitive training (treatment group, *n* = 19) or the active control condition (*n* = 19), there were no significant differences in terms of baseline demographics (Table 1) or quality of life measures (supplementary Table  3) between the two groups.

The treatment group had higher baseline cognitive scores on the BICAMS battery; however, these did not differ significantly from the control group.

The most frequently failed component of the BICAMS test battery was the SDMT with 33 (86.8%) of participants scoring below the 5th centile, 18 (47.4%) failing the CVLT-II, and 13 (34.2%) failing the BVMT-R. Overall 21 (55.2%) failed one test, 10 (26.3%) failed two tests, and 7 (18.4%) failed all three tests of the BICAMS test battery. This level of impairment is consistent with other published MS samples on BICAMS [31–33].

Overall, 88.9% of patients (16/18) in the intervention group completed at least 75% of the prescribed sessions with 66.7% (12/18) completing all the prescribed sessions.

*Behavioural Outcomes*. The main behavioural outcomes are shown in Table 2.

**Figure 1:**
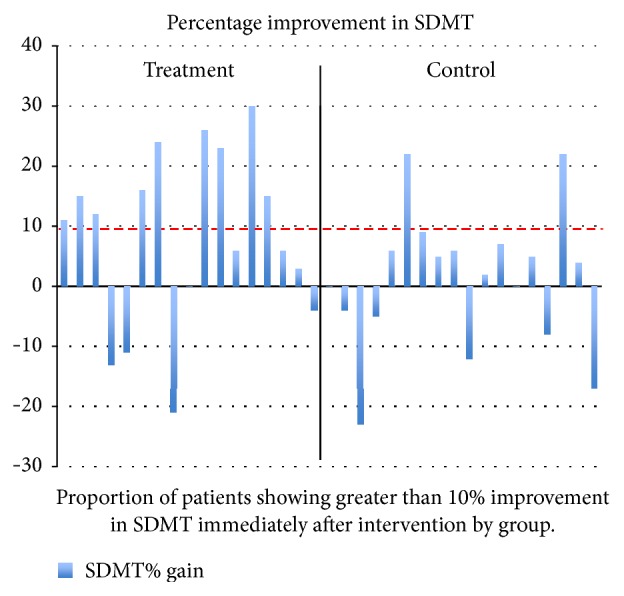
Improvement in SDMT slope immediately after intervention.

*Time 2 versus Time 1*. Compared to time 1, the treatment group showed a significantly greater improvement in gain scores between baseline and early follow-up (time 2) compared to the control group on the SDMT (treatment 3.94 (SD 5.08), controls −0.63 (SD 3.30), 1.47 to 7.66, (95% CI 1.47 to 7.66),* p* = 0.005) illustrated in Figure 1.

Similar gain scores in the CVLT and BVMT-R were not significantly different between the groups although the BVMT-R gain scores did approach significance (*p* = 0.098).

*Cognitive Outcomes: Time 3 versus Time 1*. Overall, there was an improvement in BICAMS performance across participants at follow-up. The gain scores between the groups at time 3 compared to baseline were, however, not statistically significantly different.

*QOL Outcomes*. At time 2 and time 3 there were no significant differences in QOL outcome measures, measures of self-efficacy, or subjective cognitive performance between the two groups (supplementary Table  4).

*N-Back Outcomes*. The baseline error rate between the treatment and control groups was low (8.64% versus 9.48%,* p* = 0.814). No significant differences were observed in the error rate during the *n*-back task between the groups at baseline or at follow-up (supplementary Table  5).

*Functional MRI*

*Baseline: Main Effect of Task*. The *n*-back task was associated with robust activations of several cortical areas. The 1-back task was associated with activations involving the dorsolateral prefrontal cortex bilaterally as well as bilateral inferior parietal lobule and insular and cerebellar regions relative to the 0-back contrast. The same regions were activated in the 2-back condition but the spatial extent and magnitude of the responses were greater, particularly over the frontoparietal regions (supplementary Figure  2 and supplementary Table  2).

*Time 2 versus Time 1*. At time 2, increased activation was seen in the right temporoparietal regions (right supramarginal and angular gyri (*p* < 0.005_FWE  corrected_ *at cluster level  *(*k* = 228))) in the 1-back in the treatment group relative to controls (group-by-time interaction). No significant change was seen in the 2-back task.

**Figure 2:**
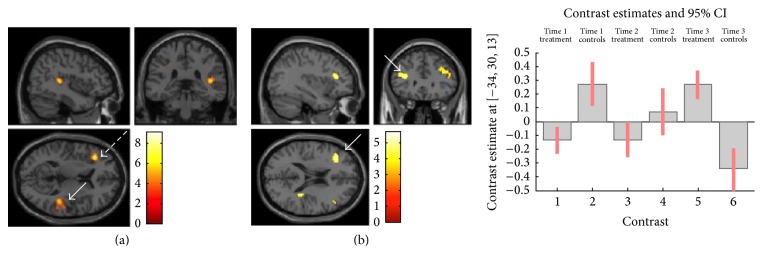
Increased activations in treatment group relative to controls at follow-up. (a) Increased activation in treatment group in right parietal region (white arrow, *p* < 0.012_FWE  corrected_) and left prefrontal region (dashed arrow, *p* < 0.001_FWE  corrected_). (b) Bilateral frontal gyrus activation in treatment group relative to controls. Left MFG activation (arrow) significant at *p* ≤ 0.042_FWE  corrected_ *at cluster level *(*k* = 152).

*Time 3*. At time 3 significant increases in activation were seen in both the 1-back and 2-back conditions in the treatment group relative to controls. In the 1-back task, increased activation was seen in the left frontal (*p* < 0.001_FWE  corrected_ *at cluster level  *(*k* = 294)) and right temporoparietal regions (*p* < 0.012_FWE  corrected_ *at cluster level  *(*k* = 187)). In the 2-back task, increases in activation were seen in bilateral prefrontal (*p* < 0.013_FWE  corrected_ *at cluster level  *(*k* = 206)) and right temporoparietal regions (*p* < 0.024_FWE  corrected_ *at cluster level  *(*k* = 178)) (Figure 2).

*Quantitative Magnetisation Transfer*. No significant between-group changes were seen in the QMT at time 2 or time 3, with respect to time 1. Overall QMT measures showed stability across all participants over the course of the study in measures of all indices.

## 4. Discussion

In line with previous work [8], the main outcome of this study was that 6 weeks of computerised cognitive rehabilitation was associated with improvement in cognitive performance as measured on the SDMT. Significant alterations in brain fMRI activations during the *n*-back task were also seen at follow-up. The SDMT improvement in the treatment group was, however, not maintained after cessation of cognitive rehabilitation (time 3 assessments) although the functional MRI changes were seen to persist at follow-up.

The SDMT is among the most sensitive tests of slowed information processing speed in MS [34, 35] and may also be a proxy for general cognitive impairment [36]. Compared to time 1, the treatment group showed a significantly greater improvement in gain scores between baseline and early follow-up (time 2) compared to the control group on the SDMT (*p* = 0.005). However, the gain scores between the groups at time 3 compared to baseline were not statistically significantly different. Overall, there was an improvement in BICAMS performance across participants at follow-up. It may be the case that cognitive rehabilitation does indeed result in improved cognitive performance but that maintenance of such improvement requires some form of ongoing intervention in the longer term. The optimum frequency and duration of cognitive training therefore remain unclear.

Clearly, repeat testing is potentially associated with practice effects. This may be particularly problematic when using the same form of a test. Only one version of the BICAMS test battery has been validated in MS and thus was used in this study. Reported test-retest coefficients on the BICAMS tests are excellent, suggesting that practice effects are negligible [31, 32]. In addition, the experimental design was to compare two groups with identical testing schedules; therefore the impact of practice effects on the results is likely to be minimal.

QOL measures did not differ significantly between groups. QOL is a complex construct influenced by a multitude of factors such as employment status, social networks, and perceptions of self-worth and self-efficacy. It is possible that cognitive rehabilitation has a positive impact on a number of these factors but such changes in such factors may take time to manifest as improvements in QOL. Further longitudinal analysis may be required to investigate this.

In order to minimise practice effects associated with repeat testing, participants were not directly trained in the *n*-back task; rather it was utilised as an outcome measure of working memory. It was anticipated that if cognitive rehabilitation was effective at improving working memory and attention, then the effects would be reflected on the performance on the *n*-back task. No differences were seen in the error rate between the groups during the *n*-back task at follow-up; however, the error rate was low at baseline in both groups.

The *n*-back fMRI paradigm in our study cohort was, however, associated with robust baseline cortical activations (in particular within the DLPFC and posterior parietal cortex) in keeping with known working memory networks [37]. A significant group-by-time interaction was seen with the treatment group exhibiting increased activation in the bilateral prefrontal cortex and right temporoparietal regions relative to control group at time 3 (*p* < 0.05_FWEcorr_).

Changes in functional activation within these regions within the treatment group are felt to be functionally relevant with respect to cognitive rehabilitation. It has been shown that the prefrontal cortex is critical in the executive control of working memory and has a role in response inhibition [38, 39]. Effective organisation of working memory may attenuate task difficulty resulting in improved working memory performance [38].

A right hemisphere dominant “ventral attentional network” consisting of the temporoparietal junction, ventral prefrontal cortex, and anterior insula is thought to be responsible for directing attention to salient events [40]. Previous work in MS has indicated that attention may be one of the domains most amenable to rehabilitation [8]. Many of the computer-training tasks involve sustained attention and it might be postulated that the increased activation seen in the right temporoparietal region at follow-up in the treatment group is as a result of improved efficiency of this network.

Interestingly, the evolution of much activity on fMRI developed after cessation of the active intervention phase. It is likely the case that solidification of neural networks occurs with training. This solidification of neural networks may extend to areas/networks outside those directly trained and may explain why working memory centres such as the prefrontal cortex were seen to be persistently active after cessation of formal training [41]. Debate remains however as to the possible interplay between adaptive and maladaptive responses during functional brain reorganisation [14].

The discrepancy between the apparent lack of clinical difference between the groups at time 3 and the sustained fMRI effect at time 3 may reflect the fact that BICAMS does not adequately measure working memory which is primarily domain utilised during the *n*-back fMRI paradigm.

Some studies have identified structural changes on diffusion tensor imaging as a result of rehabilitation in the context of physiotherapy [42, 43]. Our study attempted to explore the role of myelin in rehabilitation and repair. We did not detect any structural change on QMT after training. Due to the short duration of follow-up, this is not entirely surprising. Functional alterations in cortical activity may subsequently modulate brain structure at the microstructural level but such changes in structural brain architecture might only be detectable over the longer term.

In contrast to many previous studies, which often rely on one-to-one or outpatient administered cognitive rehabilitation, this study sought to explore whether a home-based approach to cognitive rehabilitation was feasible. The compliance rate of those undertaking the rehabilitation was excellent. A home-based approach to cognitive rehabilitation is significantly less resource intensive and may pave the way to greater access for a greater number of patients to such interventions in the future.

*Limitations*. This work has some limitations. Firstly, the groups were relatively small and there was a dropout of patients mainly in the control group between time 2 and time 3. There was heterogeneity with regard to the cognitive domains that showed deficits among participants in the study. It is likely therefore that they may not have benefited from the rehabilitation in the same way. Unfortunately the sample size of the study is too small to perform subgroup analysis.

As the study was largely exploratory in nature, it utilised an open design and is therefore subject to a number of limitations inherent to this type of design. For pragmatic reasons blinding of the investigating neurologist was not established due to the potential need for interaction between patient and investigator. This does present the potential for observer bias, particularly where repeat testing is required.

SPM analysis of MRI data offers objective, largely automated measures, which are independent of measurement bias. Investigator blinding was maintained for any methods such as assessment of white matter lesion volumes that involved manual interpretation.

In many respects, the SDMT may provide a proxy for overall cognitive functioning [44] but a more detailed cognitive assessment of the domains directly trained may have provided additional insight into effectiveness of cognitive training. BICAMS is primarily designed as a screening tool for cognitive impairment in MS assessing a limited number of domains. However, strong ecological validity has been demonstrated in relation to everyday task performance and employment, suggesting that the three domains are strongly predictive of comprehensive real-world performance [45, 46]. BICAMS may not necessarily be sensitive to change over the short-term, although the reported test-retest coefficients are excellent which would suggest sensitivity over this period [31, 32, 47].

It is postulated that alterations in fMRI activity result from microstructural changes. The lack of significant change in QMT measurements, however, suggests that the microstructural changes thought to underpin adaptive responses may, at present, be beyond the resolution of even the most advanced MRI techniques or not manifest within the timescale of this study. Additional follow-up of this cohort is planned to determine what, if any, changes are observed in terms of both cortical activation as measured by fMRI and structural changes measurable with QMT. Longer-term studies may also provide insight into the true functional impact of cognitive rehabilitation such as maintenance of employment.

## Supplementary Material

Supplementary figure 1: CONSORT flow diagram.
Supplementary Figure 2: Task main effects for all subjects.
Supplementary Table 2: Task main effects for all subjects.
Supplementary Table 3: Baseline quality of life and other behavioural measures.
Supplementary Table 4: Quality of life outcomes at follow up by treatment group.
Supplementary Table 5: N-back error rate.
Appendix for MRI methodology.

## Figures and Tables

**Table 1:** Baseline demographic characteristics and cognitive performance.

	Treatment group (*n* = 19)	Control group (*n* = 19)	Mean difference (95% CI)	*p*
	Mean (SD)	Mean (SD)		
Age (years)	46.21 (6.59)	48.53 (9.63)	−2.31 (−7.75 to 3.12)	0.588
Disease duration (years)	10.53 (6.13)	12.68 (9.87)	−2.16 (−7.56 to 3.25)	0.424
EDSS	4.42 (1.75)	4.45 (1.77)	−0.26 (−1.18 to 1.13)	0.964
Education (years)	14.05 (2.76)	13.63 (2.89)	0.42 (−1.43 to 2.28)	0.649
SDMT	43.39 (7.39)	38.21 (11.39)	5.18 (−1.27 to 11.63)	0.112
CLVT	45.32 (9.56)	43.89 (9.73)	1.42 (−4.93 to 7.77)	0.653
BVMT	20.63 (5.77)	18.05 (7.37)	2.58 (−1.77 to 6.93)	0.237

	*N*/19 (%)	*N*/19 (%)	Odds ratio (95% CI)	*p*

Gender (female)	13 (68.4)	14 (73.6)	0.74 (0.19 to 3.15)	0.721
Unemployed	13 (68.4)	11 (57.9)	1.58 (0.42 to 5.95)	0.501
Disease subtype				
Relapsing-remitting	14 (73.6)	13 (68.4)	1.29 (0.32 to 5.28)	0.721
Secondary-progressive	5 (26.3)	6 (31.6)		
On treatment at enrolment	12 (63.2)	8 (42.1)	2.38 (0.64 to 8.68)	0.194
Interferon^*∗*^	5	2		
Fingolimod	5	1		
Natalizumab	2	4		
Teriflunomide	0	1		

*∗* Includes Interferon (IF)-1b SC, IF-1A IM and IF-1A SC.

**Table 2:** BICAMS outcomes in treatment versus control groups.

	Treatment (*n* = 17)	Control (*n* = 18)	Mean difference	95% CI	*p*
	Mean (SD)	Mean (SD)			

*BICAMS improvement at follow-up (time 2 versus time 1)*					
SDMT gain	3.94 (5.08)	−0.63 (3.30)	4.56	1.47 to 7.66	0.005
CVLT gain	6.67 (7.56)	4.06 (10.10)	2.71	−3.45 to 8.87	0.377
BVMT gain	4.65 (5.18)	1.94 (4.17)	2.70	−0.52 to 5.93	0.098

	Treatment (*n* = 17)	Control (*n* = 14)	Mean difference	95% CI	*p*
	Mean (SD)	Mean (SD)			

*BICAMS improvement at follow-up (time 3 versus time 1)*					
SDMT gain	3.35 (4.17)	4.57 (7.21)	−1.28	−5.45 to 3.01	0.582
CVLT gain	6.94 (7.01)	7.50 (8.83)	−0.56	−6.38 to 5.26	0.849
BVMT gain	7.29 (5.07)	4.14 (5.32)	3.15	−0.68 to 6.98	0.105

SDMT: Symbol Digits Modalities Test; CVLT: California Verbal Learning Test; BVMT: Brief Visuospatial Memory Test.
