# Supplementary material for: A Randomised Controlled Trial of Efficacy of Cognitive Rehabilitation in Multiple Sclerosis: A Cognitive, Behavioural, and MRI Study
Source: Neural Plast. 2016 Dec 27;2016:4292585. doi: 10.1155/2016/4292585 (PMC5223046; doi:10.1155/2016/4292585)
Supplement: Supplementary file 1 — Supplementary figure 1: CONSORT flow diagram. Supplementary Figure 2: Task main effects for all subjects. Supplementary Table 2: Task main effects for all subjects. Supplementary Table 3: Baseline quality of life and other behavioural measures. Supplementary Table 4: Quality of life outcomes at follow up by treatment group. Supplementary Table 5: N-back error rate. Appendix for MRI methodology. [file 4292585.f1.pdf]

Supplemental materials

Supplementary figure 1

CONSORT flow diagram

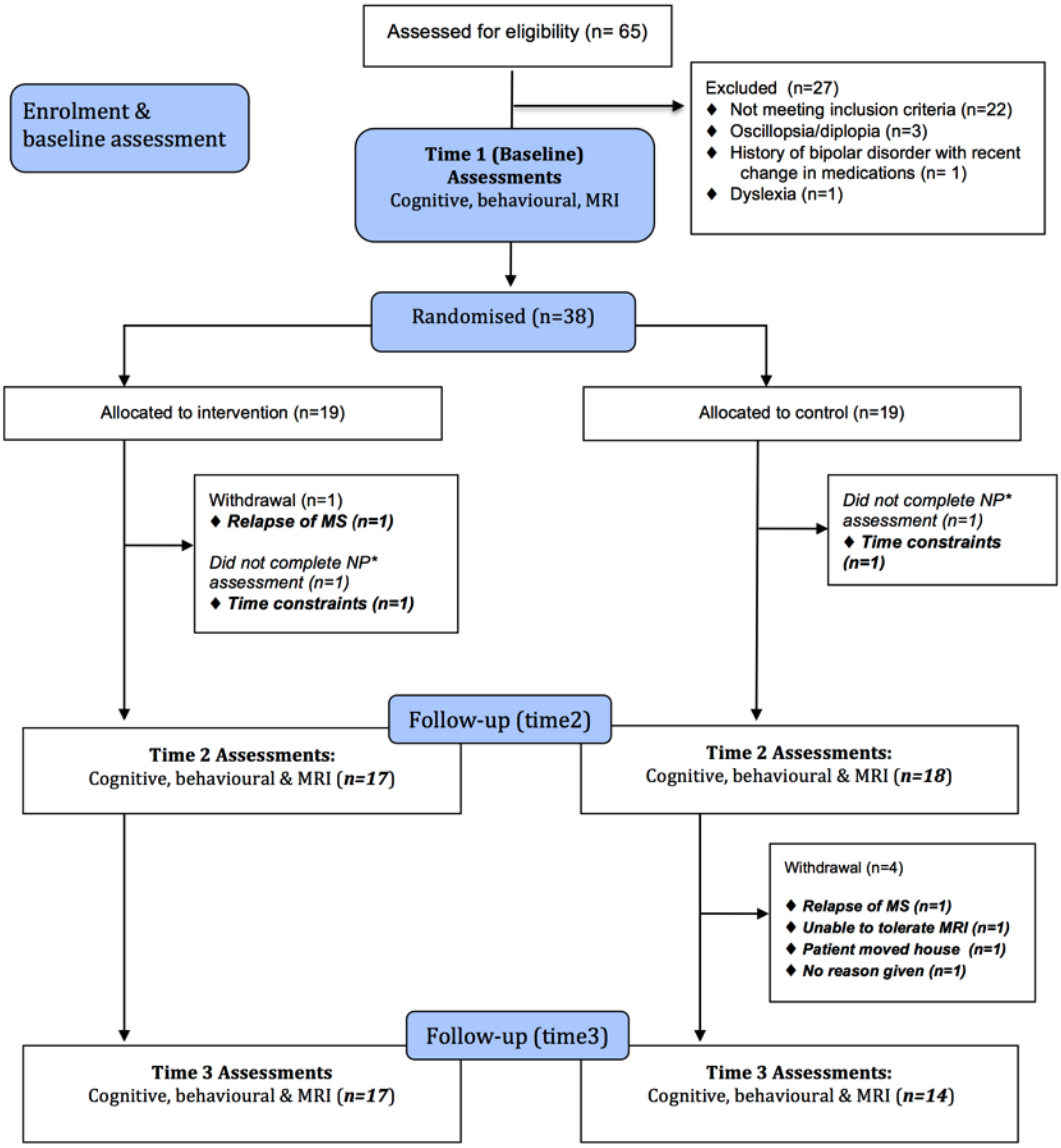

*NP: neuropsychological

Supplemental material

Supplementary figure 2: Task main effects for all subjects

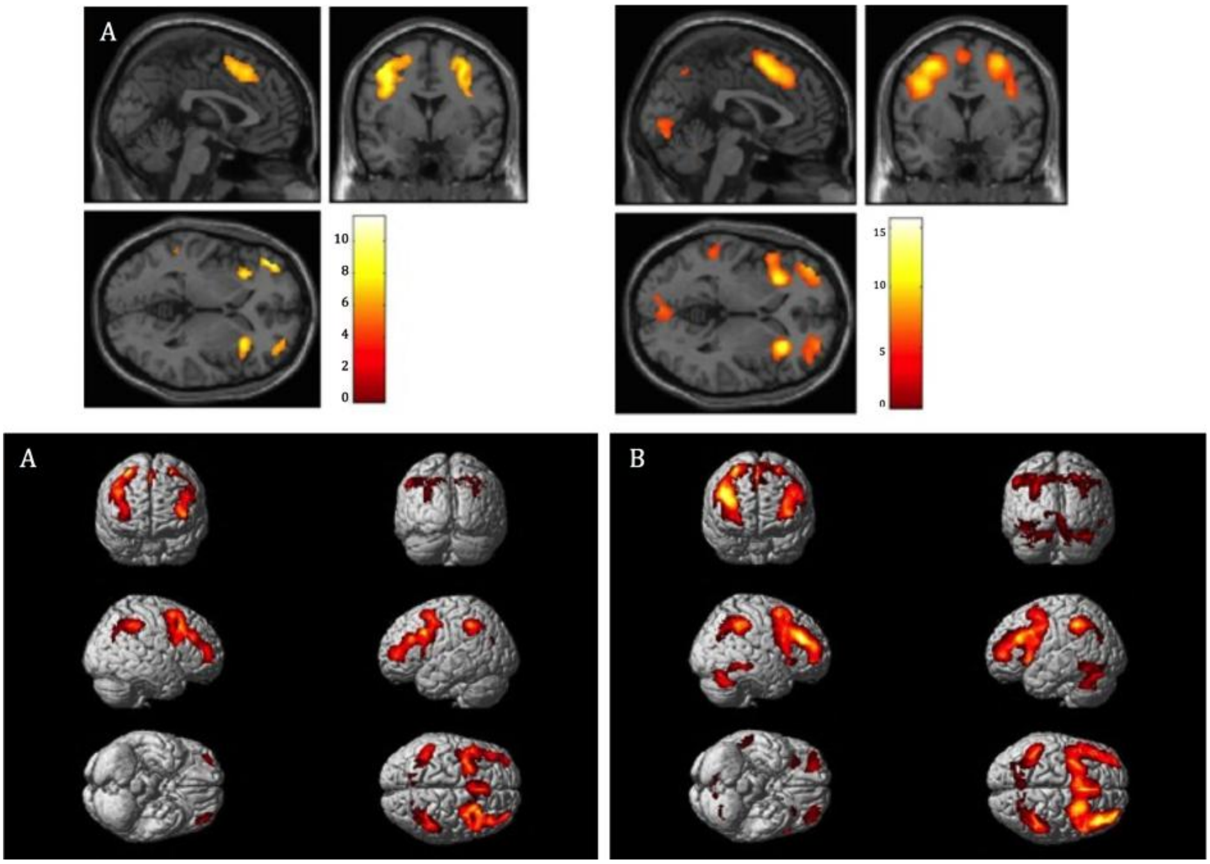

Task main effects for all subjects at baseline (n=38) showing areas of significant activation in: (a) 1-back, (b) 2-back (p <0.05 $_{\text{FWE corrected}}$).

Significant activation within prefrontal cortex (DLPFC), insula, inferior parietal lobules and occipital cortices.

Supplemental Table 2a:

Task Main effects for all subjects (baseline comparison):

Areas of Significant Activation ($p < 0.05_{\text{FWE corrected}}$) for 1-back> 0-back

**Brain region**	*MNI Coordinates*				
	x	***y***	***z***	***Max t-score***	***K_e cluster***
Dorsolateral prefrontal					
Right middle frontal gyrus	28	8	58	11.49	4258
Left middle frontal gyrus	-40	48	2	9.6	3267
Left precentral gyrus	-36	0	42	9.6	
Parietal (inferior parietal lobule)					
Right angular gyrus	34	-56	44	11.21	1940
Right supramarginal gyrus	50	-40	42	10.82	
Left supramarginal gyrus	-44	-46	46	10.27	1528
Occipital					
Right superior occipital gyrus (lateral occipital cortex)	30	-64	38	11.56	1940
Left superior occipital gyrus (lateral occipital cortex)	-24	-64	44	9.43	1528
Insula					
Left insula	-30	22	2	8.54	147
Right insula	34	22	0	8.52	234
Cerebellum					
Left Cerebellum (crus)	-26	-64	-30	7.6	210
Right Cerebellum (crus)	30	-62	-32	7.51	344

Supplemental Table 2b:

Task Main effects for all subjects (baseline comparison):

Areas of Significant Baseline Activation ($p < 0.05_{\text{FWE corrected}}$) for 2-back>0 back task

	MNI Coordinates				
**Brain region**	***x***	***y***	***z***	***Max t-score***	***K_e cluster***
Dorsolateral prefrontal					
Right middle frontal gyrus	44	34	28	15.72	25383
Left inferior frontal gyrus (pars triangularis)	-46	30	28	12.72	
Lateral Parietal (inferior parietal lobule)					
Right supramarginal gyrus	40	-44	38	13.93	6009
Left supramarginal gyrus	-38	-44	40	13.57	
Lateral Occipital					
Left middle occipital gyrus	-26	-68	36	13.19	6009
Right occipital fusiform gyrus	38	-70	-20	8.83	
Cerebellum					
Left cerebellum	-26	-66	-30	11.56	3873
Right cerebellum	30	-64	-30	10.88	

Supplemental Table 3:

Baseline Quality of life and other behavioural measures

	Treatment group (n=19)		Control group (n=19)		
	Mean	*± SD*	*Mean ±*	*± SD*	*p*
FAMS	87.26	23.00	101.06	31.98	.170
PAM-13	59.52	18.42	64.26	15.65	.398
EQ5D	0.52	0.18	0.61	0.19	0.18
USE-MS	48.26	18.01	59.74	19.96	.270
MSNQ-S	36.89	13.49	34.68	11.51	.590
HADS-D	9.47	3.55	8.47	3.21	.507
HADS-A	9.26	3.72	9.37	5.56	.946
FSS	52.37	10.40	48.84	13.59	.375

FAMS Functional Assessment of MS, MSNQ Multiple Sclerosis Neuropsychological Questionnaire,
PAM-13, Patient Activation Measure *EQ-5D,* EuroQOL five dimension questionnaire
FSS Fatigue Severity Scale
USE-MS Unidimensional Self-Efficacy scale for MS,
HADS, Hospital Anxiety and Depression Scale (HADS-D = depression, HADS-A = anxiety subscale)

Supplemental Table 4: Quality of Life outcomes at follow up by treatment group.

	**Treatment (n=17)**		**Control (n=14)**		
Scores at Time 1, Time 2 & Time 3	*Mean*	*± SD*	*Mean*	*± SD*	*p*
FAMS 1	85.24	22.61	102.79	35.06	
FAMS 2	82.18	25.09	99.14	36.89	0.719
FAMS 3	89.00	30.99	101.00	32.40	
PAM-13 1	54.62	17.13	65.58	14.66	
PAM-13 2	59.15	13.65	64.15	13.81	0.158
PAM-13 3	58.79	15.52	62.10	15.90	
EQ-5D 1	0.49	0.13	0.61	0.22	
EQ-5D 2	0.49	0.23	0.53	0.26	0.388
EQ-5D 3	0.53	0.20	0.57	0.27	
MSNQ-S 1	35.65	13.56	34.79	12.34	
MSNQ-S 2	32.76	13.81	31.36	15.83	0.892
MSNQ-S 3	29.18	15.14	28.93	13.13	
HADS-D 1	9.82	3.38	9.21	3.38	
HADS-D 2	9.82	3.34	9.50	4.35	0.921
HADS-D 3	9.35	2.85	8.79	4.21	
HADS-A 1	9.18	3.80	9.86	5.74	
HADS-A 2	9.29	4.47	8.21	5.06	0.334
HADS-A 3	8.53	4.38	6.86	4.93	
FSS 1	52.12	10.89	49.43	14.18	
FSS 2	52.53	13.21	48.57	15.64	0.912
FSS 3	52.53	11.47	49.29	15.50	
USE-MS 1	16.00	5.85	19.00	6.72	
USE-MS 2	15.76	5.62	18.69	6.60	0.996
USE-MS 3	16.47	5.70	19.31	8.70	

FAMS: Functional Assessment of MS; PAM-13: Patient Activation Measure; EQ5D: EuroQOL five dimension questionnaire; USE-MS: Unidimensional Self-Efficacy scale for MS; MSNQ: Multiple Sclerosis Neuropsychological Questionnaire; HADS-D: Hospital Anxiety and Depression Scale (depression); HADS-A: Hospital Anxiety and Depression Scale (anxiety); FSS: Fatigue Severity Scale;

Supplemental Table 5: N-back error rate

	**Treatment (n=17)**			**Control (n=14)**			
N-back Errors (Time 1, Time 2 & Time 3)	*Mean*	*SD*	*% error rate*	*Mean*	*SD*	*% error rate*	*p*
0-back 1	3.24	3.419	5.14	3.71	4.92	5.89	.951
0-back 2	1.88	2.395	6.06	2.43	3.13	7.84	
0-back 3	2.53	3.642	8.16	2.64	2.50	8.52	
1-back 1	2.71	3.478	4.30	4.57	8.42	7.25	.270
1-back 2	1.47	2.183	4.74	1.86	1.61	6.00	
1-back 3	3.06	3.269	9.87	2.14	1.99	6.42	
2-back 1	5.24	4.816	8.32	6.00	9.83	9.52	.900
2-back 2	4.12	3.551	13.29	5.29	4.68	17.06	
2-back 3	4.76	5.761	15.35	5.29	3.83	17.06	

Appendix for MRI methodology

Hardware	
Field strength	1.5T
Manufacturer	Siemens
Model	Avanto
Coil type<br>(e.g. head, surface)	32-channel head coil
Number of coil channels	32

Acquisition sequence		
Type<br>(e.g. FLAIR, DIR, DTI, fMRI)	Dual echo TSE	
Acquisition time	4 min	
Orientation	Axial-oblique	
Alignment<br>(e.g. anterior commissure/poster commissure line)	Anterior commissure-posterior commissure (AC-PC)	
Voxel size	0.898x0.898x5 mm^3	
TR	3040ms	
TE	11/ms	
TI	N/A	
Flip angle	150 degrees	
NEX	2	
Field of view	230x201mm^2	
Matrix size	256x224	
Parallel imaging		No
Cardiac gating		No
Contrast enhancement		No

Acquisition sequence	
Other parameters:<br><br>ETL<br>Number of slices	 6<br>33

Acquisition sequence		
Type<br>(e.g. FLAIR, DIR, DTI, fMRI)	Task-based fMRI<br>(ECHO planar imaging)	
Acquisition time	3 runs (9 mins each)	
Orientation	axial	
Alignment<br>(e.g. anterior commissure/poster commissure line)	AC-PC	
TR	2520ms	
TE	43ms	
TI		
Flip angle	90	
NEX		
Field of view	192x192mm2	
Matrix size	64x64	
Parallel imaging	**Yes**	
If used, parallel imaging method:<br>(e.g. SENSE, GRAPPA)	GRAPPA, acceleration factor=2	
Cardiac gating		No
Contrast enhancement		No
**Acquisition sequence**		
Type<br>(e.g. FLAIR, DIR, DTI, fMRI)	MT-bSSFP	
Acquisition time	12 volumes for a total duration of ~ 7minutes	
Orientation	Axial oblique	

Acquisition sequence		
Alignment<br>(e.g. anterior commissure/poster commissure line)	AC-PC	
Voxel size	0.93x0.93x5 mm^3	
TR	Ranging from 3.66 to 5.96 ms	
TE	=TR/2	
TI	N/A	
Flip angle	Ranging from 5 to 40 degrees	
NEX	1	
Field of view	240x180mm^2	
Matrix size	256 x 96	
Parallel imaging		No
Cardiac gating		No
Contrast enhancement		No
